# Polymer–Metal Bilayer with Alkoxy Groups for Antibacterial Improvement

**DOI:** 10.3390/polym16040508

**Published:** 2024-02-13

**Authors:** Hazem Idriss, Anna Kutová, Silvie Rimpelová, Roman Elashnikov, Zdeňka Kolská, Oleksiy Lyutakov, Václav Švorčík, Nikola Slepičková Kasálková, Petr Slepička

**Affiliations:** 1 Department of Solid-State Engineering, University of Chemistry and Technology Prague, Technická 3, 166 28 Prague, Czech Republic; 2 Department of Biochemistry and Microbiology, University of Chemistry and Technology Prague, Technická 3, 166 28 Prague, Czech Republic; 3 Faculty of Science, J. E. Purkyně University, 400 96 Usti nad Labem, Czech Republic

**Keywords:** polymer layer, PEG coating, alkoxy amine, titanium, stainless steel, diazotization, grafting, bacterial adhesion, medical devices

## Abstract

Many bio-applicable materials, medical devices, and prosthetics combine both polymer and metal components to benefit from their complementary properties. This goal is normally achieved by their mechanical bonding or casting only. Here, we report an alternative easy method for the chemical grafting of a polymer on the surfaces of a metal or metal alloys using alkoxy amine salt as a coupling agent. The surface morphology of the created composites was studied by various microscopy methods, and their surface area and porosity were determined by adsorption/desorption nitrogen isotherms. The surface chemical composition was also examined by various spectroscopy techniques and electrokinetic analysis. The distribution of elements on the surface was determined, and the successful bonding of the metal/alloys on one side with the polymer on the other by alkoxy amine was confirmed. The composites show significantly increased hydrophilicity, reliable chemical stability of the bonding, even interaction with solvent for thirty cycles, and up to 95% less bacterial adhesion for the modified samples in comparison with pristine samples, i.e., characteristics that are promising for their application in the biomedical field, such as for implants, prosthetics, etc. All this uses universal, two-step procedures with minimal use of energy and the possibility of production on a mass scale.

## 1. Introduction

Metals and metal alloys have been used in medicine for at least 5000 years [1], and they have been irreplaceable for many years for their excellent mechanical properties, biocompatibility (in the case of noble metals such as gold) or bioactivity (e.g., the possibility to achieve osseointegration in the case of titanium alloys). However, the main problems with metal prostheses were their bacterial attachment and colonization [2]. A bacteria-contaminated implants can cause, for example, acute cellular rejection [3]. As an alternative, in the second half of the 20th century, polymers were introduced into medicine and pharmacy. They brought along revolutionary solutions to the treatment of various disorders and diseases and helped in prosthetics [4,5], drug delivery [6,7], and wound healing [8].

Today, new polymers are continually being produced with better characteristics including outstanding and tissue-like mechanical properties [9,10] and excellent biocompatibility [11,12]. Their high plasticity (shaping as demanded) arises from the preparation methods, such as heat-driven polymerization [13] or usage of three-dimensional printers [14], by which the monomer is put into the meld of a desired shape before its polymerization. 

Composites consisting of metallic and polymeric parts with significantly enhanced properties, such as a perfect balance between flexibility and high toughness of the material [15], open new applications, e.g., in a trans-tissue (bone and ligaments) region [16,17]. Conventionally, composites are created using “mechanical” methods, consisting of smelting and casting, or polymer interlocking during polymerization. Such operations are typically performed under elevated temperatures or via exothermic reactions that are highly power-consuming, expensive, and unstable in certain cases. Also, there is always a possibility of generating internal or thermal stresses in the metals or unintended changes in their original bio-characteristics [18]. Hence, new methods of preparing such composites are still being sought.

One of them may consist of the covalent bond between these two materials: a metal and a polymer. Many previous works showed that functional groups of some compounds have a high potential to achieve bonding between materials of several types [19]. A reduction in these functional groups using a variety of processes (e.g., spontaneous or electrochemical diazotization) will create radicals, which can be utilized for covalent binding of the rest of the compound onto the surfaces of almost any solid material [20,21,22,23]. 

Several studies on the chemical bonding of metals and polymers have been published up to now. Alageel et al. [24] presented a method for the chemical binding of Ti to poly (methyl methacrylate) using aryldiazonium salt (*p*-phenylenediamine) as a coupling agent. This procedure leads to an excellent enhancement in the mechanical properties of this composite compared with the reference samples. Related results were obtained via a different pathway on alloy/polymer composite prepared by grafting the surface of polyamide 6 polymer with 4-nitrobenezenediazonium (NBD) salt using L-ascorbic acid (LAA). In this way, an intermediary organic layer was created, leading to an enhancement in the mechanical properties of the resulting composite and an increase in its hydrophilicity [25]. Zheng et al. [26] described a successful bioinspired bridging between Li_7_La_3_Zr_2_O_12_ alloy nanofibers and poly (ethylene oxide) by using an azole-containing compound (Dynasylan Imeo). Although this method showed significant improvements in the characteristics of the resulting materials (mainly in tensile strength), this approach has limitations due to its dependence on the chemistry of the alloy of choice, which can decrease the universality and versatility of this method and prevent it from being applied over a long line of other alloys or metals. However, the chemical stability of the bonding needs is worthy of further study since “unbinding” can happen easily in environments common in biomaterial applications.

In this work, we present a low-cost and facile chemical approach for bonding metals/alloys with polymers via an alkoxy-functional group (R-O-R’) as an intermediary agent. The method leads to a composite material with increased surface hydrophilicity, chemically stable bonding, and enhanced bacterial anti-adhesive properties. The obtained results demonstrated that the alkoxy group is a favorable candidate for achieving the coupling of metals/alloys with polymers and the utilization of the advantageous properties of both. The prepared composites could find applications in new, previously unexplored biomedicine fields. The main advantage of this method is the ability to apply it theoretically to any metal/alloy and polymers because it does not depend on their chemistry but on the properties of the alkoxy amine.

## 2. Materials and Methods

To achieve the maximum usability of the resulting composite material in various applications, a relevant metal/alloy and polymer had to be chosen. Considering the possibility of integrating the properties of both, we selected foils of titanium (99.6%, 0.5 mm, Goodfellow, Huntingdon, UK) as the metal and foils of stainless steel (SS) AISI 316 (0.5 mm, Goodfellow, Huntingdon, UK) as the metal alloy. Both types of foils were cut into 10 × 10 mm^2^ samples and cleaned by ultrasound in acetone and deionized water for 15 min each. These samples were split into two groups: (i) pristine samples, to study the characterization and functionality before grafting and modification, and (ii) samples intended for grafting and modification.

As an additive polymer layer, polyethylene glycol (PEG) was selected for its variety of applications and facile manipulation. We used PEG (Goodfellow, Huntingdon, UK) of different molecular weights, specifically, liquid (Mn of 400) and crystals (Mn of 6000), that were dissolved in methanol to reach a concentration of moles of polymeric chains of 1.3 × 10^−2^ mol/L and stirred in ultrasound until the solution was fully transparent and homogenous.

### 2.1. Salt Preparation and Reduction

For the grafting process, alkoxy amine was prepared according to the method described earlier [27]. The salt reduction process started with dissolving 0.07 g of *p*-toluene sulfonic acid (P-TSOH) in 1 mL of acetic acid (CH_3_COOH) at 25 °C. After complete dissolving, 0.03 mL of *tert*-butyl nitrite was added gradually to the mixture and stirred with a magnetic stirrer until a homogenous solution was formed. After that, 2.5 × 10^−3^ g of the alkoxy amine was added to the solution and stirred for 40 min. Finally, diethyl ether was used and added to the solution with a ratio of 1:1 and centrifuged at a speed of 7500 rpm for 30 min until full decantation of the salt. Then, the salt was washed and centrifuged one more time with diethyl ether. The remaining salt is reduced alkoxy amine diethyl (1-((1-(4-amino phenyl) ethoxy) (tert-butyl) amino)-2,2-dimethyl propyl) phosphonate.

### 2.2. Chemical Grafting

An aqueous solution of the reduced alkoxy amine with a concentration of 5700 mol/m^3^ was prepared, into which the pristine samples of Ti and stainless steel were immersed for 30 min. at room temperature 25 °C. Subsequently, the samples were washed with deionized water (3×) and then with 98% (*v/v*) methanol (3×).

### 2.3. Surface Modification

**Figure 1:**
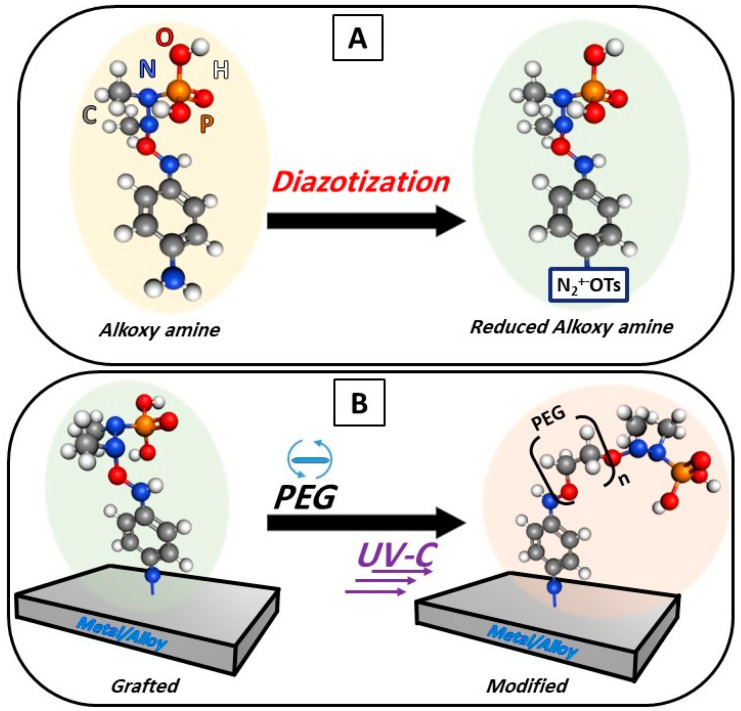
Schematic representation of salt diazotization: formation of alkoxy amine groups on the surfaces (**A**) followed by surface grafting and modification of the surface with PEG (**B**).

The polymer solutions were deposited on the surface of the grafted samples by a spin coater (1000 rpm, 1 min) to produce a homogeneous polymer layer of unified thickness. To initiate coupling, a source of UV-C light (280 nm, with an intensity of 2000 mW/cm^2^) was used for 1 h at a distance of 2 cm from the samples. The chemical grafting and surface modification steps are demonstrated in Figure 1.

### 2.4. Methods of Characterization

To confirm the chemical bonding between the surface of metal/alloys and the organic layer on one side and then between the organic layer with the polymer on the second side, Raman spectroscopy was measured on a portable ProRaman-L spectrometer (laser power 15 mW) Raman spectrometer with 785 nm excitation wavelength. Spectra were measured 30 times, each of them with a 3 s accumulation time to detect the vibrational modes of the molecules involved. Further, the surface-enhanced Raman spectroscopy (SERS) technique was used to generate a peak distribution map of the activated and reduced salt on the surface of the grafted samples. The usage of the SERS technique was chosen for the mapping due to the high accuracy of detection (single-molecule level) to give a precise distribution map of the activated, reduced, and grafted alkoxy amine, for which the usage of standard Raman is not able to constrict and to stack the spectra accurately at this stage of the modification process.

Changes in the surface roughness (*R*_a_) of pristine samples (SS or Ti) before and after grafting and the deposition of the polymer were measured by a confocal laser scanning microscope (CLSM; Olympus OLS 3100) using a magnification lens (×50) without optical zoom. Roughness was calculated using automatic calibration; it was collected from 10 different positions, and then the average was calculated to acquire accurate roughness on a micron scale.

The electrokinetic potential of all samples was determined by a SurPASS instrument (Anton Paar, Graz, Austria) in a cell with an adjustable gap at 25 °C and a pH of 6.7. The experimental error of the measurement was ±5%. For the determination of the zeta potential, the streaming current method and the Helmholtz–Smoluchowski equation were used. Specific surface area and pore volume were determined from nitrogen adsorption/desorption isotherms with a NOVA3200 (Quantachrome Instruments, Boynton Beach, FL, USA) using NovaWin software. The samples were degassed for 24 h at 60 °C. Brunauer−Emmett−Teller (BET) analysis was applied to determine the total surface area, and the Barrett−Joyner−Halenda (BJH) model was applied to determine pore volume.

The surface structure was studied using scanning electron microscopy (SEM) (Tescan Lyra 3 GMU) with a high voltage of 20.0 kV and 4.15 KX magnification. The elemental composition was measured by an energy dispersive spectroscopy (EDS) analyzer (X-MaxN) with a 20 mm^2^ SDD detector (Oxford Instruments, Abingdon, UK) embedded with the basic (SEM) device and evaluated using AZtecEnergy software to determine the spatial distribution of the elements and their approximate concentrations in the samples.

X-ray photoelectron spectroscopy (XPS) was performed using an Omicron Nanotechnology ESCAProbeP spectrometer fitted with a monochromated Al K Alpha X-ray source working at 1486.6 eV to study the composition, and these results were also used to determine the thickness of the layer (organic layer + polymer), as demonstrated before [28]. For this purpose, the well-known equation *I*/*I*_0_ = exp(−*d*/*λ* sin*θ*) was used, where *d* is the layer thickness, *λ* is the free path of the photoelectron in the organic layer, the angle *θ* with respect to the plain surface is 90°, and *I*/*I*_0_ is the ratio of detected intensities of the elements Fe/Ti before and after the modification.

Sample functionalities were extensively studied, starting with their surface wettability, which is supposed to be radically shifted after the polymer application and by the electrokinetic analyses. The wettability test consist of measuring the static and dynamic water contact angle (WCA) by goniometer Krüss (DSA 100). For the static angle, deionized water droplets with a volume of 2 μL were dropped on 10 different positions on the surface of both the pristine and modified samples. The dynamic CA measurements were performed by deposition of the water drop on the sample surface, followed by increasing and subsequently decreasing the water drop volume, varying between values of 2^−10^ μL with a speed increase/decrease of 0.03 mL·min^−1^, with simultaneous measurements of the contact angle.

Wettability tests were also used to evaluate the chemical stability of the bond. Two sets of samples were prepared (i) the first one consisting of modified samples of titanium or stainless steel as aforementioned and (ii) the second one of titanium or stainless steel with a deposited layer of the polymer modified under the same conditions but without the grafting step on the surface (i.e., metal/alloy with deposited PEG only). Both sample sets were soaked in deionized water for 30 min, air-dried, and measured by the goniometer one more time. The changes in WCA were measured at various positions and repeatedly 30 times. The establishment of chemical bonding will protect the polymer from being dissolved in water and will keep the WCA stable at hydrophilic values. In the case of missing chemical bonding, the polymer dissolves, and the goniometer reading should indicate significant changes in WCA.

Further, we studied the bacterial anti-adhesive activity of the prepared materials. For this purpose, two bacterial strains were chosen: *Escherichia coli* (*E. coli*, DBM 3138) as a model of *Gram-negative* bacteria and *Staphylococcus epidermidis* (*S. epidermidis*, DBM 2124) as a model of *Gram-positive* bacteria. Both bacterial strains were from the microorganism collection of the Department of Biochemistry and Microbiology at the UCT Prague (Czech Republic). To assess the bacterial anti-adhesive potential of the prepared materials, we used the drop plate method similarly as reported in [29,30]. First, Luria−Bertani (LB) liquid medium was inoculated with one colony-forming unit (CFU) of either *E. coli* or *S. epidermidis* [31,32] and cultivated in an orbital shaker at 120 rpm at 37 °C for 18 h. Then, the inocula were diluted in phosphate-buffered saline (PBS, sterile) at pH = 7.4 to achieve three concentrations, i.e., 8 × 10^8^, 4 × 10^7^, and 1 × 10^4^ of *E. coli* and *S. epidermidis* per mL. The evaluated samples were immersed into the bacterial suspensions and evaluated in triplicates. Then, the samples were incubated at room temperature for 1, 4, and 24 h while gently shaking. After these time points, 25 μL aliquots in five technical replicates were taken from each sample (after gentle mixing), loaded onto pre-dried LB agar plates, and incubated at 37 °C for 24 h. As a control, bacteria cultivated only in PBS, without a material sample addition, were used as well as bacterial suspensions cultivated with pristine Ti and pristine SS. After the 24-h incubation, the numbers of CFU of each bacterial strain were counted using Image J software.

In addition, to examine the bacteria incubated with the evaluated materials in greater detail, an SEM analysis of the samples was performed. After 24 h of incubation, the samples were gently washed with 1 mL of PBS and then fixed using the mixture of 2% formaldehyde with 2.5% glutaraldehyde in PBS for 4 h at 23 °C. After that, the samples were gently washed with PBS two times and then dehydrated similarly as described in refs. [33,34]. Briefly, the samples were incubated with ethanol solutions in water for 10 min in the following order: ethanol: water (*v/v*): 50, 60, 70, 80, 90, and 98%. After that, hexamethyl disiloxane (Sigma Aldrich, St. Louis, MO, USA) was added for 10 min twice and then removed, and then the samples were dried at 37 °C for 16 h. This process was followed by the sputtering of a 5 nm platinum layer and then examination by (SEM) at 10 different positions.

## 3. Results and Discussion

The process of the chemical coupling of titanium (Ti) or stainless steel (SS) with PEG can be described as a two-step procedure: (i) the reduction of the salt, as demonstrated, left free radicals, which allows the salt to be grafted on the surfaces of any solid material (here, Ti and SS), and (ii) the deposition of the PEG and then using UV-C led to the homolysis of the N-O bond of the salt, which forms radicals allowing for the bonding of the alkoxy amine and both PEG 400 and PEG 6000. However, the WCA measurements of PEG 6000 showed a more hydrophilic character; therefore, the following measurements are focused on PEG 6000.

**Figure 2:**
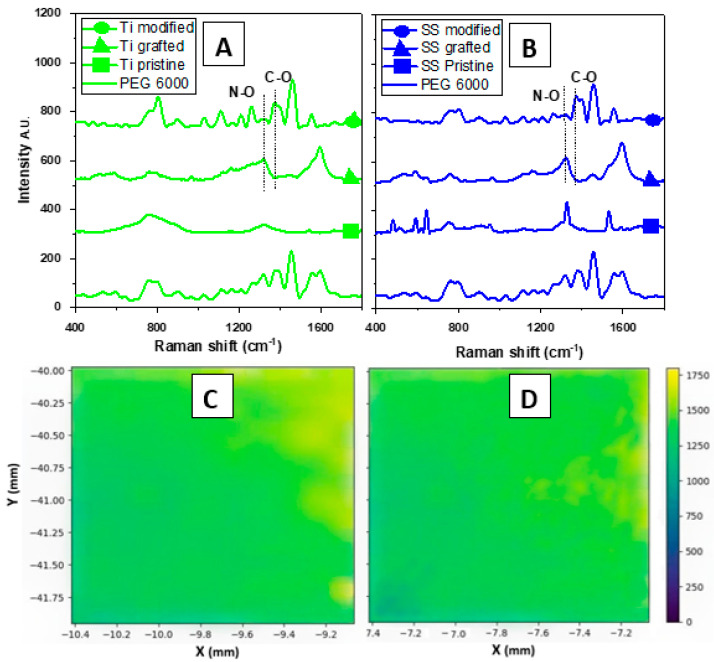
The Raman spectra of pristine, grafted, and modified Ti (**A**) and SS (**B**) samples, and Raman mapping of the significant peaks of alkoxyamine on the surface of the samples determined by the SERS technique for Ti (**C**) and SS (**D**).

The successful grafting of the salt was confirmed by Raman spectroscopy with new peaks appearing in the gathered spectra. These new peaks are found at Raman shift 450–500 cm^−1^ and 1400 and 1600 cm^−1^ for the aromatic ring, 550–600 cm^−1^, 700 cm^−1^, and 1300 cm^−1^ for Isopropyl (i-pr), 800–900 cm^−1^ and 1300 cm^−1^ for N-O, 1100 cm^−1^ and 1320–1350 cm^−1^ for P=O, and finally, at 1200 cm^−1^ for C-N [35]. Also, the successful modification with PEG 6000 can be confirmed by the changes in certain peaks, related to the interaction of PEG 6000 with the salt (see Figure 2A,B). These are related to the break of the N-O bond in the alkoxy amine, the disappearance of the peak at 1300 cm^−1^, and the formation of a new C-O bond between 1350 and 1400 cm^−1^ between PEG and alkoxy amine. Moreover, the homogenous distribution of the activated reduced salt on the surface of the stainless steel and titanium was confirmed by SERS (see Figure 2C,D).

The chemical composition of the samples before and after the modification was studied by EDX and XPS techniques. The results are presented in Table 1, where corresponding results for the concentrations of substrate elements (Fe, Cr, Ti, etc.) are shown. Both methods confirmed a dramatic increase in the concentrations of the alkoxy amine groups and the elements of the PEG 6000 polymer such as C, O, and N on the surfaces after the modification for both of the substrates, with the biggest portion of the increase being for C (between 4 and 8%), O (4–6%), and N (1–2%), respectively, which correspond with the ratios of these atoms within the formula of PEG. It is also possible to notice a decrease in the elements forming the substrates only after the modification as the penetration of the analysis is reduced because of the organic + polymer extra layer. It can also be seen that the percentage of elements forming PEG 6000 is higher for XPS than for EDX due to the depth of analysis achievable by both individual techniques.

To calculate the thickness of the layer (d), we first calculated the value of λ, which was deduced from the empirical formula derived by Seah and Dench: λ_k_ = A_n_/E^2^_K_ + B_n_/E^1/2^_K_^.^, where E_k_ is the kinetic energy of photoelectrons.

After the calculations, it was found that the thickness of the organic/polymer bonded with the Ti/SS layer was d ≈ 2.8 nm and d ≈ 2.7 nm, respectively.

**Figure 3:**
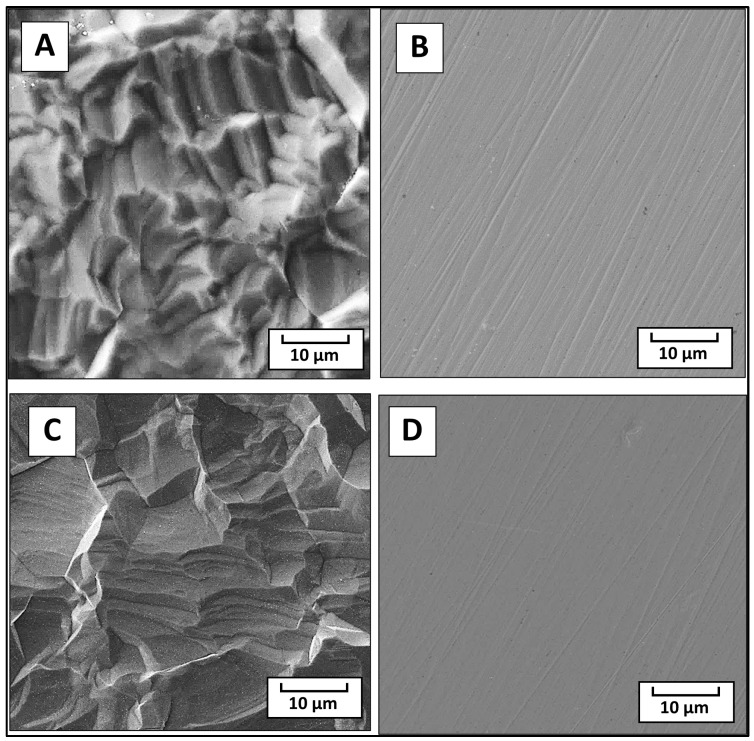
The surface morphology of the samples as observed by SEM for pristine Ti (**A**), pristine SS (**B**), Ti modified (**C**), and SS modified (**D**).

The data gathered from the CLSM showed that the surface roughness *R*_a_ of both the pristine and modified samples for SS stayed almost unchanged at an *R*_a_ of ca. 1.4 μm to 1.3 μm after the modification. For the Ti samples, however, the surface roughness slightly decreased after the modification from an *R*_a_ of ca. 1.8 to 1.2 μm. However, no significant changes in the surface morphology were observed by the SEM technique, with minor changes noticed on the original topography of the Ti (see Figure 3A for pristine Ti and Figure 3C for modified Ti). In the case of stainless steel, differences between the fusion lines by their number and depth (Figure 3B) were noticed, while the modified sample had fewer of these lines with obviously less depth (Figure 3D).

The changes in the surface roughness of the samples before and after the modification were proven also from the surface area and porosity values determined by adsorption/desorption nitrogen isotherms, as presented in Table 2. The surface area and porosity increased after the modification of both stainless steel and Ti, with more apparent changes in the Ti samples.

Changes in surface chemistry and polarity were confirmed also by electrokinetic analysis. The values of zeta potential are presented in Table 2 (the last row). These results are quite interesting, especially after the modification, when the zeta potentials changed differently for both substrates: SS and Ti. For SS, the zeta potential changed to lower negative values, from −51.3 ± 2.8 mV for the pristine SS surface to −39.0 ± 2.3 mV for the modified one, which indicates some positively charged groups on the surface (presence of amino groups) [36]. The Ti surface, however, after the modification showed more negative values of zeta potential −52.1 ± 5.6 mV in comparison with −45.7 ± 1.4 mV for the pristine Ti surface. The change can be affirmed by more negatively charged oxygen atoms on the modified surface of Ti. The diazotization process has the potential to introduce negatively charged groups onto the surface of titanium (Ti). During diazotization, a diazonium salt is typically formed, and the resulting diazonium group can be covalently attached to the Ti surface. The presence of nitrogen in the diazonium group, especially in the form of a negatively charged nitrosonium ion (NO^+^), can contribute to the overall charge of the modified surface. As was reported previously, some compounds bond to different surfaces differently, with a preferential orientation of some functional groups depending on the polar/unipolar behavior or different roughness of substrate surfaces [36]. These results can be confirmed by XPS measurement (Table 1). Here, it is clear that the amount of N atoms is 0 at unmodified samples (both Ti and SS), while after modification, the amount of N atoms is visible and is slightly higher at modified SS. Therefore, this means a higher amount of amino groups on the surface at SS, which resulted in less negative zeta potential at the modified SS in comparison with the modified Ti. We can also see the amount of oxygen atoms (O amount), which has an impact on the negative surface charge. It is clear that a higher amount of O was determined in both cases at the Ti samples. Therefore, the zeta potential is much more negative for the Ti samples (the pristine and modified ones) in comparison with the SS samples.

**Figure 4:**
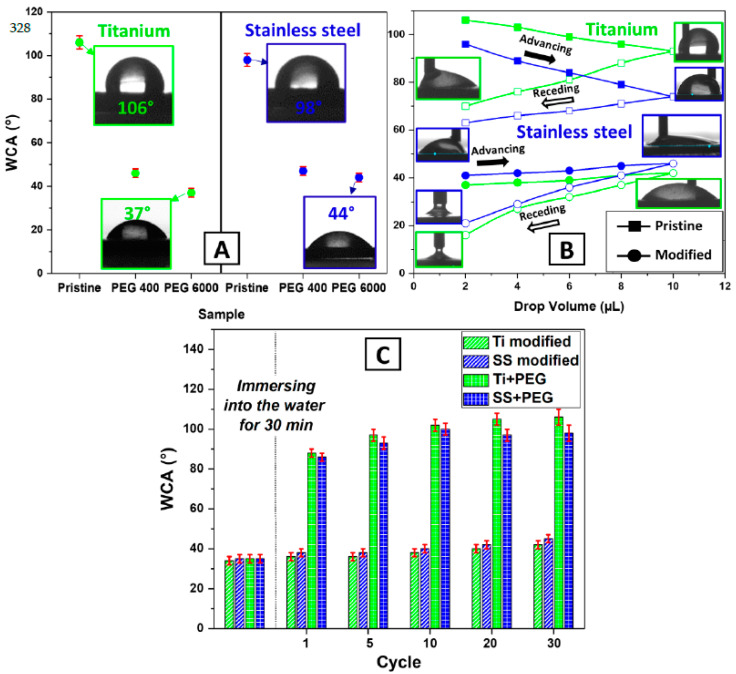
Static water contact angle (**A**); dynamic water contact angle (WCA) determined for individual samples by goniometry (**B**); and chemical stability measured by changes in WCA after immersion of the samples into water and measuring the WCA (**C**).

The modification of the samples led to an increase in the wettability of the surfaces of the composites, manifested by a lower contact angle, i.e., the WCA. This was confirmed using both dynamic and static WCA goniometry. As can be seen from Figure 4A, the static WCA significantly decreased after the material modifications, where the decrease was especially large for PEG 6000, consisting of longer chains in comparison with PEG 400. Also, the dynamic WCA results correspond with those obtained for the static WCA; the hydrophilic surface was kept at similar values whether the droplet volume was advancing or receding, in contrast to the case of the more hydrophobic pristine samples. These exhibit bigger differences, which is one of the common properties of hydrophobic surfaces, see Figure 4B. These results are the same for all substrates, and they correspond well with those of the zeta potential tests.

The results of the chemical stability tests show a higher performance of the modified samples after being immersed in water. As anticipated, the immersion leads to the dissolving of the PEG deposited layer, which was not pre-grafted, leading to the instant “collapse” of the composite hydrophilic character. In the case of the modified samples with the help of the grafted salt, the PEG 6000 chains stay attached to the surface (see Figure 4C). These findings are specifically important in showing the durability of the modified composites that can survive the next step, which is the tests of their bacterial anti-adhesive properties.

**Figure 5:**
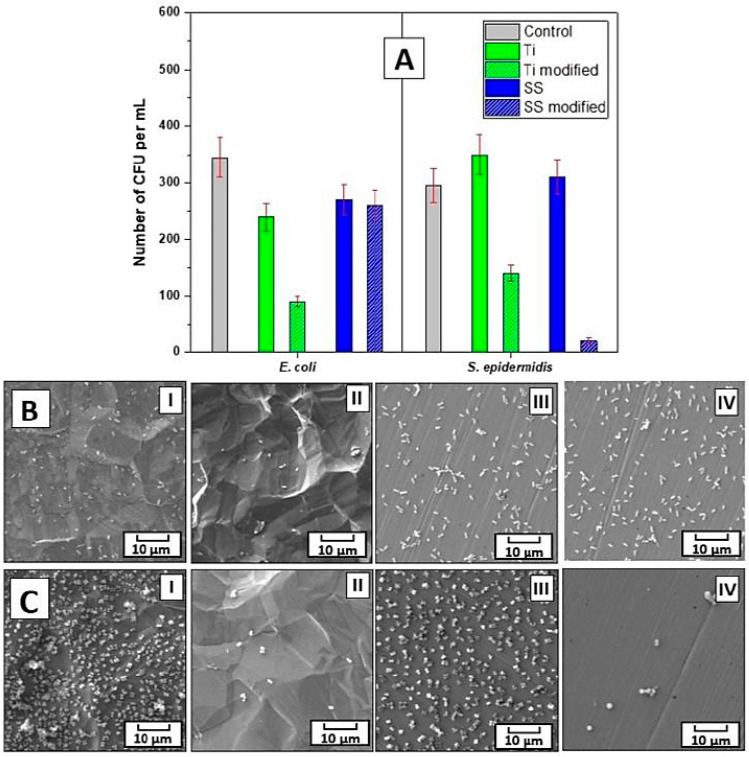
The number of colony-forming units (CFUs) of S. epidermidis and E. coli incubated for 4 h with the pristine and modified Ti and SS samples (**A**). SEM images of S. epidermidis (**B**) and *E. coli* (**C**) on the pristine and modified Ti (I and II, respectively) and the pristine and modified stainless steel (III and IV, respectively).

The bacterial anti-adhesive properties of the prepared materials were determined by CFU counting of two bacterial strains of *S. epidermidis* and *E. coli*, which were in contact with the evaluated samples for 4 h. For this purpose, three concentrations of bacterial inocula were chosen, i.e., 8 × 10^8^, 4 × 10^7^, and 1 × 10^4^ per mL. For *E. coli* at the two higher concentrations, 4 h contact with the prepared materials did not result in a decrease in the number of grown bacteria. However, for *S. epidermidis* at the 8 × 10^8^ concentration*,* there was a ca. 20% decrease in the bacteria grown when incubated with the modified Ti or SS samples, which was further pronounced to a ca. 60% decrease at the lower 4 × 10^7^ per mL concentration of bacteria for the modified SS samples in comparison with the untreated control as well as pristine samples. Notably, at the lowest concentration of bacteria, i.e., 1 × 10^4^ per mL, the modified Ti or SS impacted the number of both bacterial strains *E. coli* and *S. epidermidis*, see Figure 5A. At these conditions, there was a ca. 30% decrease in the *E. coli* CFU number after 4 h of incubation with pristine and modified SS and pristine Ti. An even more pronounced effect on the lower adhesion of bacteria was found for the modified Ti, where 4 h of contact with the bacteria resulted in a reduction in the *E. coli* CFU number by ca. 70%. Interestingly, for *S. epidermidis* at the bacteria concentration of 1 × 10^4^ per mL, the inhibition efficiency of the modified Ti and SS samples after 4 h of incubation differed from that of *E. coli*. Here, the modified SS was the most efficient in bacterial growth inhibition, the presence of which resulted in a decrease in the *S. epidermidis* CFU number by ca. 95%, while the modified Ti resulted in a decrease of ca. 50%. In contrast to the results gained for *E. coli*, no effect on the CFU number of *S. epidermidis* was detected for the pristine Ti or SS. These results were further confirmed by SEM images of the bacteria on all types of samples, see Figure 5B,C. Moreover, the data from the SEM analysis documented that only a negligible number of bacteria of both strains adhered to the modified Ti and SS samples when compared with the heavily colonized pristine samples.

The differences between the observed adhesive behavior can be understood by the original surface characteristics of the substrate (namely, surface area, porosity, and roughness), which are different between the SS and Ti samples, as shown before, and by the original biocompatibility, in addition to the way each bacterial strain adheres to the surface (surface binding with different proteins in the case of *E.coli* and *S. epidermidis*) [37,38]. However, all samples showed less adhesion of bacteria after the bonding, which means that the surface would become more bacteria-resistant, which might protect medical devices from causing infections or even the rejection of prosthetics.

These results suggest that the usage of alkoxy amine is a reliable way to create bilayer material (metal/metallic alloy and polymer) that can effectively reduce bacterial adhesion on the surface of the resulting material, which can be used for many medical devices and implants to decrease the possibility of inflammation and body rejection. When comparing it to similar approaches to form composites, whether for similar or different applications [39,40], the usage of the alkoxy amine seems to be an easier, cheaper, and more universal approach. Even though the material does not have antibacterial properties, from a chemical point of view, such properties can be acquired by including antibacterial elements (such as silver nanoparticles) [41], which can widen the application of this method considerably.

## 4. Conclusions

In this study, we demonstrated a universal, alternative approach to the conventional methods to “bond” Ti or stainless steel surfaces with PEG via the grafting of alkoxyamine. The successful chemical bonding was demonstrated by Raman measurements and changes in the element concentrations, as confirmed by EDX and XPS measurements. The successful modification resulted in significant changes in the surface properties of the materials, which were clearly shown by wettability, surface area, and zeta potential measurements. This chemical bonding technique makes it possible to fabricate composites with reliable stability in water solutions after 30 cycles and several useful functionalities. Therefore, this bonding technique deserves closer research into its applications not only in biomaterials but also in other fields. Modified Ti showed good bacterial anti-adhesive properties against both *E. coli* and *S. epidermidis*, while modified SS only showed properties against *S. epidermidis*. Therefore, both modified Ti and SS have high potential as bacterial anti-adhesive surfaces.

## Figures and Tables

**Table 1:** Element surface concentration determined by XPS and EDX for both the titanium and stainless steel pristine and modified samples.

Element	Analytic Methods			
	XPS (at. %)		EDX (at. %)	
	Titanium			
	**Pristine**	**Modified**	**Pristine**	**Modified**
**C**	35	39	4	11
**O**	40	44	3	6
**N**	0	1	0	1
**Ti**	25	16	93	82
	**Stainless steel**			
	**Pristine**	**Modified**	**Pristine**	**Modified**
**C**	48	56	8	11
**O**	28	34	1	2
**N**	0	2	0	1
**Fe**	17	5	63	61
**Cr**	4	2	17	15
**Ni**	3	1	11	10

**Table 2:** Surface area, pore volume, and electrokinetic potential (zeta potential ζ) of the individual stainless steel and titanium samples in pristine and modified forms.

	Titanium		Stainless Steel	
	Pristine	Modified	Pristine	Modified
**Surface area** (m^2^·g^−1^)	9.7 ± 2.7	14.3 ± 0.8	8.3 ± 0.1	10.9 ± 2.1
**Pore volume** (cm^3^·g^−1^)	11.0 ± 1.0	16.0 ± 0.4	9.0 ± 1.0	11.0 ± 2.0
**Zeta potential** (mV)	−45.7 ± 1.4	−52.1 ± 5.6	−51.3 ± 2.8	−39.0 ± 2.3

## Data Availability

The data presented in this study are available at https://doi.org/10.5281/zenodo.10613529 (accessed on 7 January 2024).
